# Comment on: “Effects of Plyometric Training on Physical Performance: An Umbrella Review”

**DOI:** 10.1186/s40798-023-00595-3

**Published:** 2023-08-14

**Authors:** Rodrigo Ramirez-Campillo, José Afonso, Jason Moran, David G. Behm, Urs Granacher

**Affiliations:** 1 Exercise and Rehabilitation Sciences Institute, School of Physical Therapy, Faculty of Rehabilitation Sciences, Universidad Andres Bello, 7591538 Santiago, Chile; 2 Faculty of Sport, Centre for Research, Education, Innovation, and Intervention in Sport (CIFI2D), The University of Porto, Rua Dr. Plácido Costa, 91, 4200-450 Porto, Portugal; 3 School of Sport, Rehabilitation and Exercise Sciences, University of Essex, Colchester, Essex, CO43SQ UK; 4 School of Human Kinetics and Recreation, Memorial University of Newfoundland,, St. John’s, NL Canada; 5 Department of Sport and Sport Science, Exercise and Human Movement Science, University of Freiburg, Sandfangweg 4, 79102 Freiburg i. Br., Germany

Dear Editor,

Due to the relevance of plyometric training for the strength and conditioning community and the exponential rise in the number of original research and systematic reviews on the topic, an umbrella review is needed, as it summarises findings from published meta-analyses which could meaningfully advance knowledge in this field of research. We have read with great interest the work by Kons et al. [1] entitled “*Effects of Plyometric Training on Physical Performance: An Umbrella Review*”. We understand the substantial amount of work in doing such a review (e.g. registration: December 2020; acceptance: December 2022) and acknowledge its novelty. However, some methodological shortcomings may impact their results. Our aim is to provide constructive, methodologically based criticisms so that readers have the necessary information at hand to decide if the article by Kons et al. [1] is justified or should be interpreted with a degree of caution.

*Literature search strategy.* Kons et al. [1] included 29 meta-analyses and provided a flow diagram with generic reasons for 47 excluded meta-analyses. However, and in contrast with the AMSTAR 2 recommendations [2], the authors did not incorporate a list outlining the reasons why these 47 studies were excluded. Indeed, we found 27 relevant meta-analyses [3–29] not included by Kons et al. [1]. Moreover, another eight relevant meta-analyses [30–37] were found at the time of, or after, the submission of Kons et al. [1]. Further, it is unclear why Kons et al. [1] selected one [38] above all meta-analyses related to repeated-sprint (running and cycling) training.

*Interpretation of published meta-analyses.* Kons et al. [1] concluded that:

Although several meta-analyses investigated the effects of plyometric training on physical performance outcomes, most of them lack comparisons with control groups and are classified as low-to-moderate quality.

Moreover, the authors indicated:

Five meta-analyses compared the effects of intervention to control group, while the other 24 compared within-intervention-group effects.

This seems an erroneous interpretation given that the control condition was part of the eligibility criteria for the umbrella review and, aside from one meta-analysis [39], all the meta-analyses reviewed by Kons et al. [1] performed between-group (and/or between sub-group) comparisons. Some meta-analyses compared different experimental conditions (e.g. vertical vs. horizontal jumps) or compared between-training methods (e.g. weightlifting vs. plyometric). Therefore, all outcomes analysed by Kons et al. [1] included between-group comparisons.

*Inconsistent and erroneous data.* Kons et al. [1] provided some inconsistent reporting of sample and effect sizes, heterogeneity values, among other issues (more information in Additional file 1: Table S1). For example, Kons et al. [1] reported standardised mean differences in Figs. 2–6 and classified these as trivial, small, moderate, and large. However, it is unclear how standardised mean differences were computed from meta-analyses that reported different types of effect sizes (e.g. ﻿Hedges’ g; standardised mean differences), potentially affecting Kons et al.’s [1] interpretation of their results according to the classification scheme of effect sizes.

With this letter, we encourage researchers to conduct more comprehensive umbrella reviews in the future on the effects of plyometric training on physical fitness of individuals across the lifespan.

### Supplementary Information

**Additional file 1: Table S1.** Sections from the work of Kons et al. [1] with inconsistent and erroneous data.

## Data Availability

Not applicable.

## Abbreviation

AMSTAR 2: A measurement tool to assess systematic reviews
