# Supplementary material for: Comment on: “Effects of Plyometric Training on Physical Performance: An Umbrella Review”
Source: Sports Med Open. 2023 Aug 14;9:73. doi: 10.1186/s40798-023-00595-3 (PMC10423709; doi:10.1186/s40798-023-00595-3)
Supplement: Supplementary file 1 — Additional file 1: Table S1. Sections from the work of Kons et al. [1] with inconsistent and erroneous data. [file 40798_2023_595_MOESM1_ESM.docx]

**Electronic Supplementary Material Table S1**

**Article title**: Comment on: “Effects of Plyometric Training on Physical Performance: An Umbrella Review”

**Author names**: Rodrigo Ramirez-Campillo^1^, José Afonso^2^, Jason Moran^3^, David G. Behm^4^, Urs Granacher^5^

^1^ Exercise and Rehabilitation Sciences Institute. School of Physical Therapy. Faculty of Rehabilitation Sciences. Universidad Andres Bello. Santiago, 7591538, Chile.

^2^ Centre for Research, Education, Innovation, and Intervention in Sport (CIFI_2_D), Faculty of Sport of the University of Porto, Rua Dr. Plácido Costa, 91, 4200-450 Porto, Portugal.

^3^ School of Sport, Rehabilitation and Exercise Sciences, University of Essex, Colchester, Essex CO43SQ, United Kingdom.

^4^ School of Human Kinetics and Recreation. Memorial University of Newfoundland. St. John’s, Newfoundland and Labrador, Canada.

^5^ University of Freiburg, Department of Sport and Sport Science, Exercise and Human Movement Science, Freiburg, Germany.

**Corresponding author**

Prof. Urs Granacher, PhD

University of Freiburg

Department of Sport and Sport Science

Exercise and Human Movement Science

Sandfangweg 4

79102 Freiburg i. Br.

Germany

Email: urs.granacher@sport.uni-freiburg.de

ORCID: 0000-0002-7095-813X

Table S1. Sections from the work of Kons et al. [1] with inconsistent and erroneous data.

| **Section from the work of Kons et al. [1]** | **Comment** |
| --- | --- |
| Methods | Kons et al. [1] indicated to follow recommendations (e.g., PRISMA) to conduct the umbrella review [2, 3]. We acknowledge that Kons et al. [1] used similar methods compared to a previously published umbrella review in the field [4]. However, we advise future umbrella reviews to include some key methodological steps omitted by Kons et al. [1], and advised by the aforementioned umbrella review [4] and by updated recommendations to conduct umbrella reviews [5, 6] (e.g., clear definition of comparators; usage of GRADE).  A substantial proportion of the methods from the published peer-reviewed manuscript (or pre-print [7]) were not detailed in the registered protocol (PROSPERO, CRD42020217918). If changes occurred, these should have been explicitly stated in the manuscript, as per PRISMA [3] and PRISMA 2020 statements [6].  The authors indicated:  “*We conducted a systematic literature search in the databases PubMed/MEDLINE, Scopus, SPORTDiscus, Web of Science, Cochrane Library and Scielo.*”  However, the Boolean search syntax used, provided in Appendix 1 by Kons et al. [1] indicate the following databases: PUBMED, Web of Science, Scopus, Google scholar. Additionally, it would be advisable that future umbrella reviews contact experts in the field (e.g., through Expertscape: <https://expertscape.com/go/plyometric%20exercise>) to seek potentially additional relevant studies.  It is unclear why the authors included a meta-analysis [8] related to repeated-sprint (running and cycling) training. While muscle actions during sprint running occur in the stretch shortening cycle, and could therefore be classified as plyometric activity, (repeated) sprint running is certainly not a form of plyometric jump training. Independent from conceptualization and operationalization issues, if sprint training studies were includable, it is unclear why Kons et al. [1] selected only one meta-analysis [8], considering that more sprint-related meta-analyses are available in the literature. Future umbrella reviews should provide a clear explanation (conceptually and operationally) regarding the plyometric training method under investigation.  Relatedly, the authors do not provide well-defined inclusion/exclusion criteria for the outcome category reported in Table 1. Further, the authors use the concept “physical fitness” in the inclusion criteria, although in the main text the authors use different concepts: “physical capacities”, “sports performance”, “physical performance”, “physical fitness performance”, “physical tasks”, “physical aspects”. Terminology should consistently be applied throughout the manuscript to avoid confusion. For example, in the classical (and broadly accepted) definition of physical fitness from Caspersen et al. [9], body composition-related outcomes should also be considered or explain why they examined selected physical fitness qualities only. |
| Results, Table 2, number of participants and their characteristics | For the meta-analysis of Alfaro-Jimenez et al. [10], Kons et al. [1] indicated a sample size of N = 50 participants. This seems to be a sub-estimation, as a sample size of N >800 can be derived from Table 1 in the meta-analysis of Alfaro-Jimenez et al. [10].  Similarly, for the meta-analysis of Asadi et al. [11], Kons et al. [1] indicated a sample size of N = 46. This seems to be a sub-estimation, as a cohort of N > 800 can be derived from Table 1 in the meta-analysis of Asadi et al. [11].  Regarding the comparison between weightlifting exercises vs. plyometric training in one meta-analysis [12], four studies with a total sample size of N = 76 were included, in contrast with the report by Kons et al. [1] of N = 158.  In some meta-analysis (e.g., [13]) Kons et al. [1] summarized participants from intervention and control groups as study sample (N = 129). That is correct. However, in other meta-analyses (e.g., [14, 15]), it is unclear why they decided not to report the number of participants from the control groups.  For three meta-analyses [16-18], it is unclear why Kons et al. [1] indicated between 24 to 122 participants only, since the Table 1 from each of the three meta-analyses indicates evident greater values.  In the meta-analysis of Moran et al. [19], Kons et al. [1] indicated that the number of participants was “n.r.” (i.e., not reported). However, Moran et al. [19] reported the number in Figure 3 (N = 199).  In the meta-analysis of Asadi et al. [11], Kons et al. [1] indicated that:  “*Youth athletes-practitioners and non-practitioners of sports*” were included.  However, both youth and adult participants were considered. |
| Results, Table 2, Column “Effect size (95% CI, *p* value); (*p* value)” | The authors did not provide a clear explanation regarding the meaning of the second “(p value)”. From the pre-print version [7] published by Kons et al., it seems that the second “(p value)” refers to heterogeneity. If our interpretation is correct, then several of the p values reported by Kons et al. [1] in Table 2 of the published version need a revision. For example, Kons et al. [1] appear to confuse between Egger test p values and heterogeneity results in some meta-analysis (e.g., [20]).  For Moran et al.’s meta-analysis [21], Kons and colleague [1] reported:  “*0.66 (0.33–0.98, p = 0.02); (p < 0.001)*”, although the p values do not match with those from the original meta-analysis.  In one meta-analysis [22], the authors reported effect sizes using both fixed and random model meta-analyses. It is unclear why Kons et al. [1] decided to report the values derived from the fixed model, particularly since most meta-analyses included in the umbrella review used a random model approach. Further, Kons et al. [1] reported only the results for the 20-m linear sprint test, omitting results from the 10-m and 30-m linear sprints. Further, sub-group results (e.g., participants >18 vs. <18 y of age) were not reported.  For one meta-analysis [12], Kons et al. [1] reported an I^2^ = 21% which is in contrast to the I^2^ = 0% reported in the original meta-analysis. |
| Results, Figure 2 | Kons et al. [1] indicated an unclear effect for 5-, 10-, 15-, 20-, and 30-m sprint times in male soccer players [23]. Aside from the fact that the cited meta-analysis [23] was not included in Figure 2, the “unclear” classification used by Kons et al. [1] was not explained in the manuscript. Kons et al. [1] indicated that standardised mean difference values <0.20 were classified as “trivial”. Although the authors might have confused the term “unclear” with “trivial”, this does not explain why the authors reported an unclear effect for 20-m sprint time, since the meta-analysis [23] reported a significant effect (p=0.02). Moreover, it is unclear why Kons et al. [1] reported “n.a.” (i.e., not assessed) in Table 2 in reference to the p-values of the 5-, 10-, 15-, 20-, and 30-m sprint time meta-analysis [23], since these were reported by van de Hoef et al. [23]. |
| Results, Figure 3 | It is unclear why Kons et al. [1] omitted a relevant meta-analysis [11] in Figure 3, and in the analysis-results of change-of-direction performance.  Kons et al. [1] postulated:  “*Unclear effect was observed for individual sport athletes and young athletes.*”  However, Kons et al. [1] did not explain why they used the term “unclear” for the studies [24, 25], since both achieved either small or moderate effect sizes according to the data interpretation proposed by Kons et al. [1]. Moreover, Asadi et al. [24, 25] reported significant changes.  For one meta-analyses [24], Kons et al. [1] calculated an effect size = 0.59. No clear explanation was provided by Kons et al. [1] regarding their calculation method. Moreover, sub-group effect size values (Hedge’s g values) according to participants’ age (maturity status), were not provided by Kons et al. [1]. |
| Results, Figure 5 | According to Kons et al. [1], changes on *power or explosive muscular strength performance* were represented in Figure 5. However, the meta-analysis of Alfaro-Jimenez et al. [10] investigated the effects on jump performance, and thus probably should be included in Figure 6 rather than Figure 5. |
| Results, Figure 6. | For one meta-analysis [26], Kons et al. [1] indicated an effect size of 2.07. However, the authors of the respective meta-analysis [26] emphasized that a corrected value of 0.82 (after outlier exclusion) should be considered, in line with recent recommendations [27]. It is unclear why Kons et al. [1] decided to use the uncorrected value. |
| Results, Figures 2 to 6 | Kons et al. [1] indicated:  “*The following data were extracted from the included meta-analyses:…..effect sizes and the equations used to compute effect sizes…)*.”  They also indicated:  “…*differences were found in the respective equations that were used to compute SMDs. …. Therefore, we extracted the effect sizes for each included meta-analysis*…”  However, no clear explanation was provided regarding calculations performed to compute standardised mean differences from meta-analyses that reported effect sizes other than standardised mean differences. This may impact the interpretation of findings. For example, Kons et al. [1] indicated that for change of direction performance:  “*A large effect was observed in basketball players (for running distances shorter or longer than 40 m)* [20].”  However, the “large” classification seems to be based on the Hedges’ *g* effect size values reported in the original meta-analysis [20], without correction for the standardised mean difference classification used by Kons et al. [1]. Indeed, the authors of the meta-analysis [20] indicated only a “moderate” effect. |
| Results, Figures 4, 5, and 6 | All *trivial-unclear* standardised mean differences values reported by Kons et al. [1] in Figures 4, 5, and 6 come from meta-analyses that either focused on upper-body plyometric training or compared plyometric training with weightlifting exercises [12] or traditional strength training [28]. Future umbrella reviews are advised to consider the type of comparator group and to distinguish between upper- and lower-body plyometric training in the sub-group analyses.  Relatedly, although Kons et al. [1] provided group-specific and/or outcome-specific values for some studies, such as for the meta-analysis of Behm et al. [28], providing effect size values for sub-groups such as trained and untrained boys, Kons et al. [1] did not provide effect size values for the sub-groups that were included in other studies (e.g., [24]).  Further, some meta-analyses (e.g., [20, 28]) reported several outcomes (e.g., jump; sprint), with a different number of participants included in each outcome analysis. Kons et al. [1] reported the global number of participants in the meta-analyses. Future umbrella reviews may consider reporting the exact number of participants per outcome, to assess meta-analyses on an outcome-specific basis [29]. |
| Results, Table 3 | AMSTAR 2 results may need a thorough revision based on current observations. |
| Discussion | In the opening paragraph of the discussion section, Kons et al. [1] indicated:  “*The most concerning finding from our study is the lack of control group comparisons and the low-to-moderate quality for most of the meta-analyses available in the literature.*”  Probably, statements such as this must be reviewed with caution, considering our previously discussed observations related to the work made by Kons et al. [1] regarding their assessment of control group comparisons. Further, the quality of the meta-analyses available in the literature (assessed by Kons et al. [1] in Table 3) could be re-assessed. Furthermore, most (22 of 29) of the meta-analyses assessed by Kons et al. [1] attained a moderate quality, opposed to the “low-to-moderate” classification statement made by Kons et al. [1]. |
| Conclusion | Kons et al. [1] indicate:  “*Nonetheless, it is important to bear in mind that most meta-analyses did not include a control condition, limiting the strength of some statements mentioned in papers. This systematic umbrella review unveiled an important weakness of the present research topic. Although several meta-analyses investigated the effects of plyometric training on physical performance outcomes; most of them lack comparisons with control groups and are classified as low-to-moderate quality. It is advised that the outcomes from this umbrella review must not be considered as level 1 evidence.*”  Based on our observations, the conclusion may need (major) revision. |
| References | Several references need revision in their numeration. For example, the numeration of several references appearing in Figures 2-6 do not match with the numeration provided in the reference list. |
| AMSTAR 2: a measurement tool to assess systematic reviews.  GRADE: grading of recommendations, assessment, development, and evaluations. | |

**REFERENCES**

1. Kons RL, Orssatto, L.B.R., Ache-Dias, J., De Pauw, K., Meeusen, R., Trajano, G.S., Dal Pupo, J., Detanico, D. Effects of plyometric training on physical performance: An umbrella review. Sports Med - Open. 2023;9.

2. Aromataris E, Fernandez R, Godfrey CM, Holly C, Khalil H, Tungpunkom P. Summarizing systematic reviews: Methodological development, conduct and reporting of an umbrella review approach. Int J Evid Based Healthc. 2015;13(3):132-40.

3. Liberati A, Altman DG, Tetzlaff J, Mulrow C, Gøtzsche PC, Ioannidis JPA, et al. The PRISMA statement for reporting systematic reviews and meta-analyses of studies that evaluate healthcare interventions: Explanation and elaboration. BMJ. 2009;339:b2700.

4. Lesinski M, Herz M, Schmelcher A, Granacher U. Effects of resistance training on physical fitness in healthy children and adolescents: An umbrella review. Sports Med. 2020;50(11):1901-28.

5. Belbasis L, Bellou V, Ioannidis JPA. Conducting umbrella reviews. BMJ Medicine. 2022;1(1):e000071.

6. Page MJ, McKenzie JE, Bossuyt PM, Boutron I, Hoffmann TC, Mulrow CD, et al. The PRISMA 2020 statement: An updated guideline for reporting systematic reviews. BMJ. 2021;372:n71.

7. Kons RL, Orssatto, L.B.R., Ache-Dias, J., De Pauw, K., Meeusen, R., Trajano, G.S., Dal Pupo, J., Detanico, D. Effects of plyometric training on physical performance: An umbrella review. Pre-print, SporRxiv. 2022.

8. Taylor J, Macpherson T, Spears I, Weston M. The effects of repeated-sprint training on field-based fitness measures: A meta-analysis of controlled and non-controlled trials. Sports Med. 2015;45(6):881-91.

9. Caspersen CJ, Powell KE, Christenson GM. Physical activity, exercise, and physical fitness: Definitions and distinctions for health-related research. Public Health Rep. 1985;100(2):126-31.

10. Alfaro-Jiménez D, Salicetti-Fonseca A, Jiménez-Díaz J. [Effect of plyometric training on explosive strength in team sports: a meta-analysis] [Article in Spanish]. Pensar en Movimiento: Revista de ciencias del ejercicio y la salud. 2018;16(1):e27752.

11. Asadi A, Arazi H, Young WB, Saez de Villarreal E. The effects of plyometric training on change-of-direction ability: A meta-analysis. Int J Sports Physiol Perform. 2016;11(5):563-73.

12. Berton R, Lixandrão ME, Pinto ESCM, Tricoli V. Effects of weightlifting exercise, traditional resistance and plyometric training on countermovement jump performance: A meta-analysis. J Sports Sci. 2018;36(18):2038-44.

13. Ramirez-Campillo R, Alvarez C, Garcia-Hermoso A, Keogh JWL, Garcia-Pinillos F, Pereira LA, et al. Effects of jump training on jumping performance of handball players: A systematic review with meta-analysis of randomised controlled trials. Int J Sports Sci Coach. 2020;15(4):584-94.

14. Ramirez-Campillo R, Pereira LA, Andrade D, Méndez-Rebolledo G, de la Fuente CI, Castro-Sepulveda M, et al. Tapering strategies applied to plyometric jump training: A systematic review with meta-analysis of randomized-controlled trials. J Sports Med Phys Fitness. 2021;61(1):53-62.

15. Ramirez-Campillo R, Sanchez-Sanchez J, Romero-Moraleda B, Yanci J, García-Hermoso A, Manuel Clemente F. Effects of plyometric jump training in female soccer player's vertical jump height: A systematic review with meta-analysis. J Sports Sci. 2020;38(13):1475-87.

16. de Villarreal ES, Kellis E, Kraemer WJ, Izquierdo M. Determining variables of plyometric training for improving vertical jump height performance: a meta-analysis. J Strength Cond Res. 2009;23(2):495-506.

17. Sáez de Villarreal E, Requena B, Cronin JB. The effects of plyometric training on sprint performance: A meta-analysis. J Strength Cond Res. 2012;26(2):575-84.

18. Sáez-Sáez de Villarreal E, Requena B, Newton RU. Does plyometric training improve strength performance? A meta-analysis. J Sci Med Sport. 2010;13(5):513-22.

19. Moran J, Ramirez-Campillo R, Liew B, Chaabene H, Behm DG, García-Hermoso A, et al. Effects of bilateral and unilateral resistance training on horizontally orientated movement performance: A systematic review and meta-analysis. Sports Med. 2021;51(2):225-42.

20. Ramirez-Campillo R, García-Hermoso A, Moran J, Chaabene H, Negra Y, Scanlan AT. The effects of plyometric jump training on physical fitness attributes in basketball players: A meta-analysis. J Sport Health Sci. 2022;11(6):656-70.

21. Moran J, Ramirez-Campillo R, Granacher U. Effects of jumping exercise on muscular power in older adults: A meta-analysis. Sports Med. 2018;48(12):2843-57.

22. Kayantaş I SM. Effect of plyometric training on speed parameters (a meta-analysis study). Int J Appl Exerc. 2020;9(8):117-30.

23. van de Hoef PA, Brauers JJ, van Smeden M, Backx FJG, Brink MS. The effects of lower-extremity plyometric training on soccer-specific outcomes in adult male soccer players: A systematic review and meta-analysis. Int J Sports Physiol Perform. 2020;15(1):3-17.

24. Asadi A, Arazi H, Ramirez-Campillo R, Moran J, Izquierdo M. Influence of maturation stage on agility performance gains after plyometric training: A systematic review and meta-analysis. J Strength Cond Res. 2017;31(9):2609-17.

25. Sole S, Ramírez-Campillo R, Andrade DC, Sanchez-Sanchez J. Plyometric jump training effects on the physical fitness of individual-sport athletes: A systematic review with meta-analysis. PeerJ. 2021;9:e11004-e.

26. Ramirez-Campillo R, Andrade DC, Nikolaidis PT, Moran J, Clemente FM, Chaabene H, et al. Effects of plyometric jump training on vertical jump height of volleyball players: A systematic review with meta-analysis of randomized-controlled trial. J Sports Sci Med. 2020;19:489-99.

27. Kadlec D, Sainani KL, Nimphius S. With great power comes great responsibility: Common errors in meta-analyses and meta-regressions in strength and conditioning research. Sports Med. 2023;53(2):313-25.

28. Behm DG, Young JD, Whitten JHD, Reid JC, Quigley PJ, Low J, et al. Effectiveness of traditional strength vs. power training on muscle strength, power and speed with youth: A systematic review and meta-analysis. Frontiers in physiology. 2017;8:423.

29. Guyatt GH, Oxman AD, Montori V, Vist G, Kunz R, Brozek J, et al. GRADE guidelines: 5. Rating the quality of evidence--publication bias. Journal of clinical epidemiology. 2011;64(12):1277-82.
